# Long-term potentiation at C-fibre synapses by low-level presynaptic activity *in vivo*

**DOI:** 10.1186/1744-8069-4-18

**Published:** 2008-05-28

**Authors:** Ruth Drdla, Jürgen Sandkühler

**Affiliations:** 1 Department of Neurophysiology, Center for Brain Research, Medical University of Vienna, Vienna, Austria

## Abstract

Inflammation, trauma or nerve injury trigger low-level activity in C-fibres and may cause long-lasting hyperalgesia. Long-term potentiation (LTP) at synapses of primary afferent C-fibres is considered to underlie some forms of hyperalgesia. In previous studies, high- but not low-frequency conditioning stimulation of C-fibres has, however, been used to induce LTP in pain pathways. Recently we could show that also conditioning low-frequency stimulation (LFS) at C-fibre intensity induces LTP *in vitro *as well as in the intact animal, i.e. with tonic descending inhibition fully active. In the slice preparation, this form of LTP requires a rise in postsynaptic Ca^2+^-concentration and activation of Ca^2+^-dependent signalling pathways. Here, we investigated the signalling mechanisms underlying this novel form of LTP *in vivo*. We found that the signal transduction pathways causing LFS-induced LTP *in vivo *include activation of neurokinin 1 and N-methyl-D-aspartate receptors, rise of [Ca^2+^]_i _from intracellular stores and via T-type voltage-dependent Ca^2+ ^channels, activation of phospholipase C, protein kinase C and Ca^2+^-calmodulin dependent kinase II. These pathways match those leading to hyperalgesia in behaving animals and humans. We thus propose that LTP induced by low-level activity in C-fibres may underlie some forms of hyperalgesia.

## Background

LTP at the first synapse in pain pathways is considered to underlie some forms of pain amplification e.g. after trauma, inflammation or nerve injury [1]. A strong rise in postsynaptic calcium ion concentration triggering Ca^2+^-dependent signal transduction pathways is required for LTP induction [2-4]. Consequently, high-frequency (~100 Hz), burst-like stimulation protocols were previously used to induce LTP at virtually all synapses studied so far.

Low-level activity between 1–10 imp·s^-1 ^rather than high frequency bursts are, however, typical for C-fibre discharges during inflammation, trauma or wound healing. Presynaptic activity at these low frequencies is considered inadequate to cause a sufficiently strong rise in postsynaptic [Ca^2+^]_i _for potentiation of synaptic strength. In fact, low-level presynaptic activity was either ineffective or induced synaptic long-term depression (LTD) rather than LTP in previous studies.

We have recently discovered that in a spinal cord slice preparation with long dorsal roots intact LFS of dorsal roots at C-fibre intensity induces LTP which involves a rise in postsynaptic [Ca^2+^]_i _and Ca^2+^-dependent signal transduction pathways [4]. In the intact animal spinal dorsal neurons are, however, under a powerful tonic inhibition arising from supraspinal, descending pathways [5,6]. This inhibition is inevitably lost in the *in vitro *situation and could thereby facilitate LTP-induction. And indeed, removal of descending, putatively inhibitory pathways by spinalisation is required for the induction of LTP by either pinching or noxious heating of the skin [7]. On the other hand, we have shown recently that in the intact animal, LTP can be induced by LFS as well as by subcutaneous capsaicin or formalin injections also [4]. Here, we further characterised this novel LFS-induced LTP at C-fibre synapses in the intact animal.

## Methods

### Preparation of the animals

After obtaining approval from the Institutional Animal Care Comitee (Austrian Federal Ministry for Education, Science and Culture), experiments were performed on adult male Sprague Dawley rats (200 – 300 g) obtained from a local breeding facility.

Animals were intubated using a 16 G cannula and mechanically ventilated (Siemens Servoventilator 900C). Isoflurane in two thirds N_2_O and one third O_2 _was used to induce (4 vol% inspiratory) and maintain (1.5 vol% expiratory) anaesthesia. Surgical level of anaesthesia was verified by stable arterial blood pressure and the absence of paw withdrawal reflexes after pinching the paw. Surgical procedures have been described in more detail elsewhere [4]. Briefly, the right femoral vein and artery were cannulated to allow i.v. infusions and to monitor arterial blood pressure. Muscle relaxation was achieved by 2 μg·kg^-1^·h^-1 ^pancuronium bromide i.v The left sciatic nerve was dissected free for bipolar electrical stimulation using a silver hook electrode. Lumbar segments L4 and L5 were exposed by laminectomy. The dura mater was incised and reflected. Two metal clamps were used for fixation of the vertebral column in a stereotactic frame. An agarose pool was formed around the exposed spinal segments. The spinal segments were covered with artificial cerebrospinal fluid which was carefully removed before application of drugs (see below). The exposed sciatic nerve was covered with warm paraffin oil. At the end of the experiment animals were decapitated in deep anaesthesia and the spinal cord removed to locate the recording site (see next paragraph).

### Electrophysiological recordings

Electrophysiological recordings were performed as described elsewhere [4]. Briefly, C-fibre-evoked field potentials were recorded with glass electrodes (2–3 MΩ) in laminae I and II of the spinal cord dorsal horn in response to stimulation of the ipsilateral sciatic nerve. Electrodes were driven by a microstepping motor (Narishige Scientific Instrument, Japan). Recordings were made with an ISO-DAM-amplifier (World Precision Instruments Inc., Sarasota, FL, USA) using a band width filter of 0.1 – 1000 Hz. Signals were monitored on a digital oscilloscope (Tektronix TDS 210) and digitized at a sampling rate of 5 kHz by an A/D converter (DigiData 1200 Series Interface). Field potentials may be evoked by A- or C-fibres. The different components can easily be distinguished by their thresholds and latencies. C-fibre evoked field potentials have high thresholds (> 5–6 V) and long latencies (90 – 150 ms, corresponding to the conduction velocity of < 1.2 m·s^-1^). The potentials were not abolished by spinalisation or muscle relaxation, strongly suggesting that these signals are evoked by activation of primary afferent C-fibres. This is in line with a report by Schouenborg [8], suggesting that field potentials are generated monosynaptically by synapses between primary afferent fibres and second order neurons. A polysynaptic contribution to the signals can, however, not be fully excluded. As test stimuli, single pulses (0.5 ms, 25 V, which is suprathreshold for the activation of C-fibres) were delivered to the sciatic nerve every 5 minutes using an electrical stimulator (Iso-Stim 01D, npi electronic, Germany). Stable responses for 1 hour served as control. LTP of C-fibre evoked field potentials was induced by low frequency stimulation (LFS, 2 Hz, 0.1 ms pulses, 60 V, 2 min). After conditioning stimulation, test pulses were applied again to the sciatic nerve at 5 min intervals for up to 7 hours.

Rhodamine B (0.2%, Sigma) was added to the pipette solution which consisted of (in mM) NaCl 135, KCl 5.4, CaCl_2 _1.8, Hepes 10 and MgCl_2 _1. For identification of the recording position, pressure was applied to the electrode (300 mbar, 1 min) at the end of each electrophysiological experiment. The spinal cord was removed, cryo-fixed and the rhodamine B spot was localised under a fluorescence microscope. Only those experiments where the tip of the recording electrode was located in lamina I or II were included into the study.

### Drugs

Pancuronium bromide (Pancuronium-ratiopharm^®^, Ratiopharm GmBH, Ulm, Germany) was given as an i.v. infusion (2 μg·kg^-1^·h^-1^). The competitive N-methyl-D-aspartate (NMDA) receptor antagonist D(-)-2-Amino-5-phosphonopentanoic acid (D-AP5, 100 μM, Sigma) and the T-type voltage-dependent Ca^2+ ^channel (VDCC) blocker mibefradil dihydrochloride hydrate (5 mM, Sigma) were dissolved in 0.9% NaCl. The protein kinase C (PKC) blocker chelerythrine chloride (800 μM, Sigma) was dissolved in ddH_2_O. The Ca^2+^-calmodulin dependent kinase II (CaMKII) blocker 2- [N-(2-hydroxyethyl)]-N-(4methoxybenzenesulfonyl)]amino-N-(4-chlorocinnamyl)-N-methylbenzylamine (KN-93, 400 μM, Sigma), the phospholipase C (PLC) blocker 1- [6-[((17β)-3-Methoxyestra-1,3,5[10]-trien-17-yl)amino]hexyl]-1H-pyrrole-2,5-dione (U-73122, 500 μM, Sigma), the neurokinin receptor 1 (NK1 receptor) blocker cis-2-(Diphenylmethyl)-N- [(2-iodophenyl)methyl]-1azabicyclo [2.2.2]octan-3-amine oxalate salt (L-703,606, 5 mM, Sigma) and the ryanodine receptor blocker dantrolene (500 μM, Tocris) were dissolved in DMSO. Dantrolene was further dissolved in standard solution titrated to a pH of 9.5. All other drugs were further dissolved in artificial cerebrospinal fluid to obtain desired concentrations as indicated and applied directly onto the spinal cord [9], starting 30 min prior LFS.

### Data analysis and statistics

The area under the curve of C-fibre evoked field potentials was determined offline using the software Clampfit 9.0 (Molecular Devices Inc.). For the electrophysiological experiments, responses were normalised for each rat. A one way repeated measures analysis of variance (One way RM-ANOVA) was performed to compare the different experimental protocols and treatments. ANOVA was corrected by the Bonferroni adjustment. A p-value < 0.05 was considered statistically significant. Values are expressed as mean ± standard error of mean (SEM).

## Results and discussion

**Figure 1:**
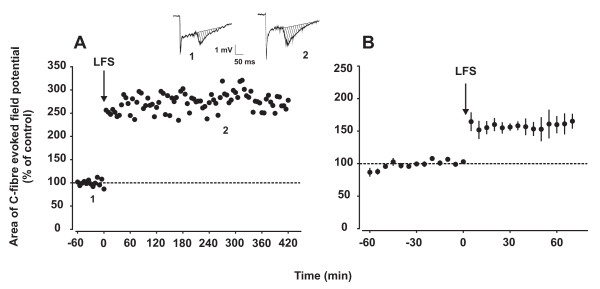
**LTP in the spinal dorsal horn is induced by LFS of C-fibres in the sciatic nerve**. Electrical LFS at C-fibre strength delivered to the sciatic nerve induced LTP of C-fibre evoked field potentials in laminae I/II of the spinal cord dorsal horn, lasting undiminished for several hours. A representative example of LFS-induced LTP and two original recordings prior to (1) or after LTP-induction (2) are shown in (A). Areas of C-fibre evoked field potentials were used to quantify synaptic strength (dashed areas in 1 and 2). Mean time course of five experiments is shown in (B). In both graphs, area of C-fibre evoked field potentials (% of control) is plotted against time (min). The arrows represent the time point of conditioning LFS.

Intact, adult rats were used in the present study to evaluate LTP induction *in vivo *by low-level activity in C-fibres. Electrical LFS (2 Hz, 2 min, C-fibre strength) delivered to the sciatic nerve induced stable LTP of C-fibre evoked field potentials always lasting until the end of the recording period of up to 7 hours (Fig. 1A). LFS-induced mean LTP of C-fibre evoked field potentials to 160% ± 13% of control (p < 0.05, n = 5) at 60 min after conditioning stimulation (Fig 1B; Table 1). A-fibre evoked field potentials were never affected (data not shown). In some subsequent experiments DMSO was used as a solvent for drugs applied directly onto the spinal cord surface. Superfusion of the spinal cord at the recording segment with this solvent had no effect on LTP induction in any of the 3 animals tested (Table 1).

**Table 1:** Summary of results

Superfusate	Concentration	C-fibre response (% control)	n
ACSF	-	160% ± 13%*	5
DMSO	25%	150% ± 14%*	3
Mibefradil	5 mM	108% ± 14% n.s.	5
L-703,606	5 mM	88% ± 15%*	5
Dantrolene	500 μM	103% ± 14% n.s.	5
U-73122	500 μM	107% ± 10% n.s.	5
Chelerythrine	800 μM	103% ± 7% n.s.	5
D-AP5	100 μM	107% ± 8% n.s	5
KN-93	400 μM	99% ± 5% n.s	5

Mean (± SEM) C-fibre evoked field potentials are expressed as % of control 60 min after LFS. One-way repeated measure-ANOVA was used to compare areas of C-fibre evoked field potentials before and after LFS. The asterisk denotes significant differences from baseline (* = p < 0.05). Drugs in spinal superfusate and their respective concentrations are indicated.

**Figure 2:**
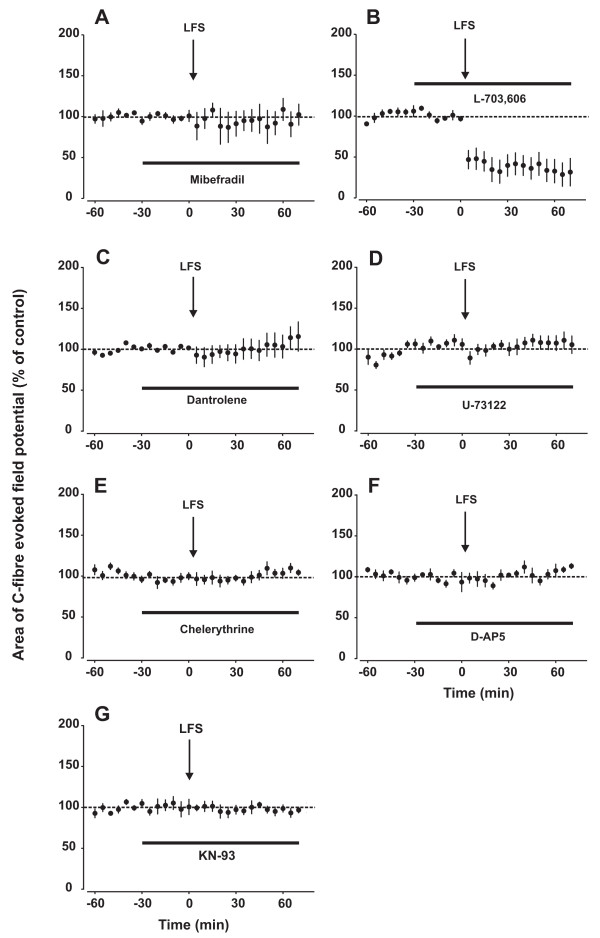
**Signalling pathways underlying the induction of LFS-induced LTP *in vivo***. In all graphs, area of C-fibre evoked field potentials (% of control) is plotted against time (min). Data are expressed as mean ± SEM (n = 5 for all groups). Spinal superfusion at the recording segment with the T-type VDCC blocker mibefradil (5 mM, A), the ryanodine receptor blocker dantrolene (500 μM, C), the PLC inhibitor U-73122 (500 μM, D), the PKC blocker chelerythrine (800 μM, E), the NMDA receptor blocker D-AP5 (100 μM, F) or the CaMKII blocker KN-93 (400 μM, G) fully blocked LTP induction by LFS. Blockade of spinal NK1 receptors with L-703,606 led to induction of LTD rather than LTP by LFS (B). See also Table 1.

We have shown previously that *in vitro *LFS-induced LTP requires a rise in [Ca^2+^]_i _[4]. This may be achieved by Ca^2+^-influx from the extracellular space or by release from intracellular compartments [10]. During LFS, voltage-dependent Ca^2+ ^channels (VDCCs) which open already by moderate depolarization, may be activated. To test if low-voltage activated T-type VDCCs play a role, we applied the blocker mibefradil (5 mM) directly onto the spinal cord 30 min prior to LFS. Topical mibefradil completely prevented LTP induction (Fig. 2A; Table 1), suggesting that LFS of sciatic C-fibres is sufficient to activate T-type VDCCs in spinal cord, and that this contributes to LTP induction. Our finding is in line with recent studies showing that spinal T-type VDCCs play a role both for normal nociception, and for thermal and mechanical hyperalgesia in models of neuropathic pain [11,12].

Upon stimulation substance P is released from C-fibre afferents, leading to activation of neurokinin-1 (NK1) receptors, mainly in laminae I/II neurons of the spinal dorsal horn [13]. Most NK1-expressing lamina I neurons have a supraspinal projection [14]. Blockade of spinal NK1 receptors attenuates thermal and mechanical hyperalgesia [15-17]. In the present study, blocking spinal NK1 receptors with L-703,606 (5 mM) not only fully blocked LTP induction but rather led to LFS-induced LTD of C-fibre evoked field potentials (Fig 2B; Table 1).

The thresholds for the induction of LTP and LTD have been suggested to be tuned narrowly. Changes in the level of elevation of [Ca^2+^]_i _might shift the threshold from LTP towards LTD [18]. The polarity of synaptic plasticity further depends upon the magnitude [19], the temporal pattern [20] and the mode of postsynaptic Ca^2+ ^elevation [21]. These parameters are all relevant for activation of distinct Ca^2+^-dependent signal transduction pathways involving protein phosphatases and kinases. Interestingly, blockade of NK1 receptors but not of VDCCs or NMDA receptors (see below) might critically change at least one of these characteristics of the rise in [Ca^2+^]_i_, shifting synaptic plasticity from LTP towards LTD. This illustrates that blocking different routes of [Ca^2+^]_i _rise may have differential effects on synaptic plasticity.

In superficial spinal dorsal horn neurons [Ca^2+^]_i _may be increased by the release of Ca^2+ ^from intracellular stores [10] via activation of either ryanodine or inositol 1,4,5-trisphosphate (IP_3_) receptors. Ryanodine receptors may be activated by Ca^2+ ^which may, for example, enter the cell through VDCCs, leading to Ca^2+^-induced Ca^2+ ^release from ryanodine sensitive stores [10]. In the present study, spinal ryanodine receptors were blocked by dantrolene (500 μM), a clinically used ryanodine receptor antagonist. This abolished LTP induction in all animals tested (Fig 2C; Table 1). Intrathecal injection of dantrolene has previously been shown to reduce nociceptive behaviour caused by spinal application of a NK1 receptor agonist [22].

Another trigger for Ca^2+ ^release from intracellular stores is the activation of IP_3 _receptors. IP_3 _receptors are activated by IP_3_, a product formed by hydrolysis of phosphatidylinositol 4,5 bisphosphate upon activation of the phosphoinosite system. It starts with activation of phospholipase C (PLC) by the alpha subunit of a G_q_-protein [23]. Activation of PLC in the spinal cord is essential for hyperalgesia. For example, mechanical hyperalgesia induced by intraplantar injection of endothelin 1, a peptide produced by many different cell types, requires activation of spinal PLC [24]. Furthermore, intrathecal pre-treatment with a PLC inhibitor reduced the second-phase in the formalin test [25]. In the present study, inhibition of PLC in spinal cord by the selective blocker U-73122 (500 μM) completely prevented induction of LTP by LFS (Fig. 2D; Table 1).

PLC activation also leads to the production diacylglycerol (DAG) which in turn activates protein kinase C (PKC). At least 12 isoforms of this enzyme can be distinguished, many of those are present in superficial laminae of the spinal cord dorsal horn [26]. PKC plays a crucial role in the development of hyperalgesia. Activation of spinal PKC by phorbol esters evokes thermal hyperalgesia and mechanical allodynia in awake rats [27]. An involvement of PKC in inflammatory pain has also been shown. For example, PKC inhibitors attenuate hyperalgesia after intradermal injection of capsaicin or formalin [28,29]. PKC may phosphorylate the NMDA receptor at distinct phosphorylation sites, thereby increasing channel activity [30,31] or removing the Mg^2+ ^block [32]. Here, blockade of spinal PKC by chelerythrine (800 μM) abolished induction of LTP by LFS in all animals tested (Fig. 2E; Table 1).

Activation of Ca^2+^-permeable NMDA receptors in spinal dorsal horn is an essential step for the induction of several forms of hyperalgesia and allodynia [33-35]. However, NMDA receptors are normally blocked near the resting membrane potential by Mg^2+^. One might speculate that LFS is not sufficient to activate NMDA receptors, unless the Mg^2+ ^block is removed e.g. by phosphorylation via PKC. And indeed, LFS-induced LTP required activation of NMDA receptors, as blockade of spinal NMDA receptors with the competitive NMDA receptor antagonist D-AP5 (100 μM) prevented LTP induction (Fig 2F; Table 1). This is similar to findings of previous studies showing that NMDA receptor activation is also required for high frequency stimulation (HFS)-induced LTP in the spinal cord dorsal horn [36-38,3].

A rise in [Ca^2+^]_i _may lead to Ca^2+ ^binding to calmodulin, which in turn activates Ca^2+^-calmodulin dependent kinase II (CaMKII). CaMKII undergoes a Ca^2+^-dependent autophosphorylation, resulting in Ca^2+^-independent activity [39]. One of its main targets is the GluR1 subunit of the AMPA receptor, which gets phosphorylated at Ser831 [40]. This leads to an increased channel conductance and thus synaptic strength [41]. Activation of spinal CaMKII is required for some forms of hyperalgesia [42-44]. For example, intrathecal pre-treatment with a CaMKII inhibitor prevents thermal hyperalgesia and mechanical allodynia after injection of complete Freund's adjuvant into a hindpaw [45]. Inhibition of CaMKII blocks induction of HFS-induced LTP in the spinal cord dorsal horn [46].

Here, the CaMKII inhibitor KN-93 (400 μM) abolished induction of LFS-induced LTP in all animals tested (Fig 2G; Table 1).

## Conclusion

In the present study, we investigated signal transduction mechanisms underlying LFS-induced LTP in the spinal cord *in vivo*. We could show that pathways required for LTP induction match those involved in hyperalgesia. This suggests that LFS-induced LTP at spinal synapses of C-fibres may be a mechanism of pain amplification in behaving animals and perhaps human subjects [47].

Conditioning electrical stimulation of C-fibres at low frequencies induces LTP at the first nociceptive synapses in the spinal cord dorsal horn in slice preparations as well as in intact animals [[4], present study]. The signal transduction pathways underlying this novel form of LTP have been previously investigated *in vitro *only [4]. Here we demonstrate that pathways underlying the induction of LFS-induced LTP *in vivo *fully match those in the *in vitro *slice preparation suggesting that the same cellular elements are involved despite the very different experimental conditions. The absence or presence of descending modulatory pathways or general anaesthesia does apparently not affect the principle mechanisms of LFS-induced synaptic plasticity in superficial spinal dorsal horn.

**Figure 3:**
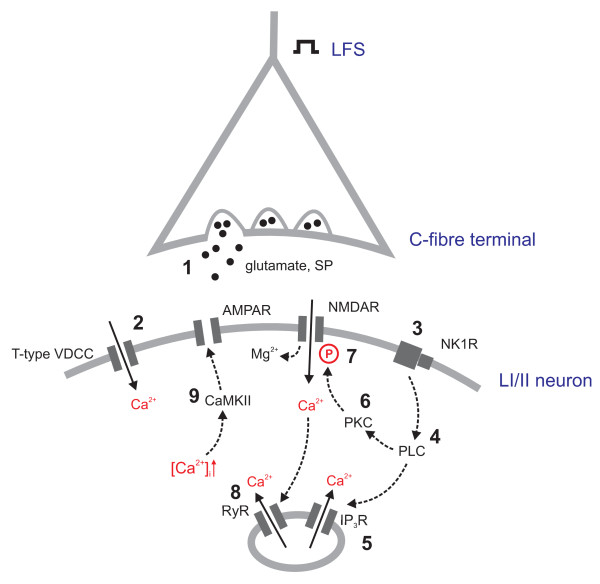
**Potential mechanisms of LTP in spinal dorsal horn *in vivo***. At synapses between C-fibres and laminae I/II second order neurons, LFS triggers the release of the excitatory neurotransmitters glutamate and substance P leading to depolarization of the postsynaptic cell (1). T-type VDCCs are activated (2), leading to Ca^2+ ^influx. The activation of NK1 receptors (3) activates the PLC-pathway (4), triggering the release of Ca^2+ ^from intracellular stores (5) and the activation of PKC (6). This kinase phosphorylates NMDA receptors, thereby loosening their Mg^2+ ^block (7). Ca^2+^-induced Ca^2+ ^release activates ryanodine receptors, thereby further increasing [Ca^2+^]_i _(8). The increased [Ca^2+^]_i _is sensed by CaMKII that phosphorylates AMPA receptors, thereby potentiating synaptic strength. LFS: low frequency stimulation; SP: substance P; VDCC: voltage-dependent Ca^2+ ^channel; AMPAR: alpha-amino-3-hydroxy-5-methyl-4-isoxazolepropionic acid receptor; NMDAR: N-methyl-D-aspartate receptor; NK1R: neurokinin receptor 1; PLC: phospholipase C; PKC: protein kinase C; IP_3_R: inositol 1,4,5-trisphosphate receptor; RyR: ryanodine receptor; CaMKII: Ca^2+^-calmodulin dependent kinase II;

Based on the results of the present study, the following sequence of events leading to the induction of LTP at synapses of primary afferent C-fibres can be suggested (Fig. 3): LFS of the sciatic nerve triggers the release of excitatory neurotransmitters including glutamate and substance P (1.), which leads to a moderate depolarization of postsynaptic neurons. This is sufficient to activate low-voltage activated T-type Ca^2+ ^channels and Ca^2+ ^influx into the cell (2.). In addition, NK1 receptors are activated (3.). Downstream to this receptor activation, the PLC-pathway (4.) leads to the release of Ca^2+ ^from intracellular stores (5.) as well as to activation of PKC (6.). This kinase phosphorylates the NMDA receptor (7.), thereby reducing its Mg^2+ ^block. By this, moderate postsynaptic depolarization may become sufficient to open NMDA receptor channels, leading to an additional influx of Ca^2+^. [Ca^2+^]_i _rise is further enhanced by Ca^2+^-induced Ca^2+ ^release from ryanodine sensitive stores (8.). CaMKII is activated by an increased [Ca^2+^]_i _and phosphorylates AMPA receptors, thereby increasing their channel conductance. Its activity is also necessary for translocation of AMPA receptors to the subsynaptic membrane [48]. This ultimately results in an increased strength of excitatory synapses in nociceptive pathways (9.) and amplification of nociception and perhaps pain perception.

## Competing interests

The authors declare that they have no competing interests.

## Authors' contributions

RD and JS participated equally in the conception, design and interpretation of the study, RD performed all experiments and data analysis and drafted the manuscript. Both authors read and approved the final manuscript.
